# Esophageal Infection of *Spirocerca lupi* with *Vulpes rueppellii* in Yazd Province, Iran: Case Report

**Published:** 2019

**Authors:** Seyed Mohammad HOSEINI, Hooman RONAGHI, Bahram Ali ZAHERI, Amir Hossein MOSHREFI, Mohammad Ali ADIBI

**Affiliations:** 1. Department of Pathobiology, Babol Branch, Islamic Azad University, Babol, Iran; 2. Department of Microbiology, Faculty of Specialized Veterinary Science, Science and Research Branch, Islamic Azad University, Tehran, Iran; 3. Department of Environmental Protection Semnan, Semnan, Iran; 4. Young Researchers and Elite Club, Babol Branch, Islamic Azad University, Babol, Iran

**Keywords:** *Spirocerca lupi*, *Vulpes rueppellii*, Esophageal infection, Iran

## Abstract

*Spirocerca lupi* is an international nematode of Canine and wild carnivores. The generic clinical symptoms are regurgitation, vomiting, and pursiness. The parasite usually procreates nodular masses in the esophagus and pectoral aorta. On July 2014, a four-month male sandy fox was found accidentally killed on the road in Bafgh City, Southeast of Yazd, Yazd Province, Iran during necropsy, referring to esophagus, parasites were collected from esophagus and transmissive up to 70% of alcohol. Parasites were recognized as *S. lupi*. After clarification of lactophenol and staining, the nematodes were recognized as *S. lupi* according to key guidelines of Taylor. Besides, the tissue samples were isolated from esophagus wall - the appendix site of nematode for histopathological investigation - so they were transmissive up to 10% of buffered formalin and stained with haematoxylin and eosin. Microscopic diagnoses in esophagus were included epithelial destruction, wall thickening, inflammatory cells infiltration, necrosis and vascular thrombosis. With the author's knowledge, this is the first report and histopathological investigation of *S. lupi* in sandy fox.

## Introduction

*Vulpes rueppellii* is known as desertic and semi-desertic areas from the Atlantic Ocean to Somalia. The absence of voluminoused and accurate reports about the existing of *Vulpes rueppellii* around the situation and ecology in its repartition range in Iran crowd remainders is almost unknown (1,2).

Spirocercosis is a parasitic disease due to *Spirocerca lupi*, the etiological factor is a nematode of the order Spirurida, family Spirocercidae, and subfamily Spirocercinae. The mature one is reddish in paint and displays a pronounced sexual difference: the female one is 6–7 cm in length whilst the male does not trespass a length of 3–4 cm (3).

*S. lupi* is a nematode that focuses on the esophageal and stomachic wall and with intervals in disordered places such as the heart, lungs, lymphoid ganglia, mediastinum, reins muscle fasciae and bladder of canines and exotic carnivores. It is found universe-wide, especially in tropical and subtropical areas. The life period of *S. lupi* contains intermediate (coprophagous beetles) and paratenic hosts (fowl, savage birds, crocodiles, rodents, porcupine and hares), infected with the larvae (L3) and the ultimate host (carnivore). Spirocercosis can have an extended width of clinical signs containing regurgitation and vomiting, coughing, faint or narcosis, and weight loss (3–5).

The adult worms are existed coiled within granulomatous nodules organized usually in the esophagus, wherever they put embryonated (L1) eggs, passed in the gastrointestinal lumen by small fistulous tracts by which the parasite has been opened via the esophageal wall. The eggs are passed in the stools or in the vomit. Spirocercosis is commonly subclinical; however, it can be the reason for esophageal/stomach granulomas and sarcomas, aortal scarring, pectoral disco spondylitis, hypertrophic osteopathy and salivary gland necrosis, too (6). In progressive cases, the lesions of spondylitis of the caudal pectoral vertebra and neoplastic has been changed to fibrosarcoma, osteosarcoma and undifferentiated sarcoma can be observed (7).

Larvae reason necrosis, bleeding, and neutrophil infiltration in the vessel wall observed, then, they migrated; otherwise, they hit for the pectoral aorta. These lesions generally healed overall. In the pectoral aorta, decadent elastic and muscle tissues become fibrotic, and sometimes it mineralized to constant appearance, intimal scars and aneurysms of change measured and numbered. The intensity of harm perhaps associated with the measure of infective larvae derived to infection period.

Esophageal tumors are uncommon in dogs and it is reported for only 0.5% of canine instances of neoplasms recognized with esophageal sarcoma (8).

The aim of the current report was to study the histopathological change with *S. lupi* in the esophagus of a sandy fox.

This *research* is the *first* study *in Yazd Province* that *describes* pathological change of *S. lupi* in the esophagus of *Vulpes rueppellii*.

## Case report

**Fig. 1:**
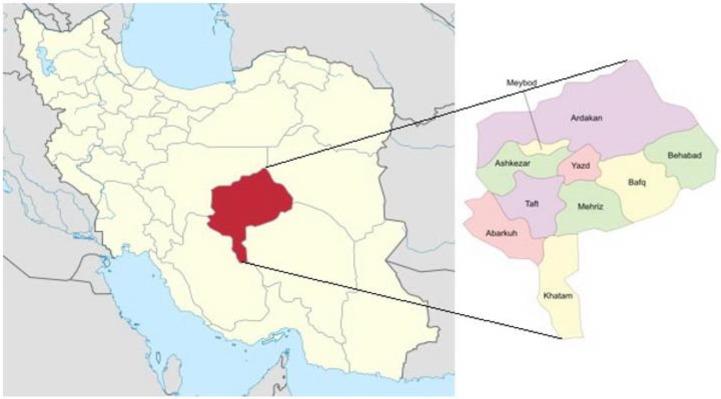
Map of Iran, the location of Bafgh County situated in Yazd Province, Iran

On July 2014, a four-month male sandy fox was found accidentally killed on the road in Bafgh City, Southeast of Yazd, Yazd Province, Iran (latitude N 31.47; longitude E 55.38) (Fig. 1). The gastrointestinal (*GI*) *tract* was transferred to the Laboratories of Veterinary Diagnostic Medicine at the Islamic Azad University of Babol Branch for histopathological examinations. Necropsy investigations were carried out with specific reference to the Esophagus. The coarse changes were recorded, and free worms were gathered and transferred into 70% of alcohol solution. After clarification by lactophenol and painting, the nematodes were identified as *S. lupi* based on applying guidelines. Tissue samples 10% of buffered formalin-fixed, paraffin-embedded Esophagus tissue of the infected Sandy Fox were created and stained by routine processes of haematoxylin and eosin (H&E) staining. Consecutive sections were incision using a rotary microtome (Leitz, 1512, Germany) at 5 μm. Then, the slides meticulously assayed by using microscopes. The study followed the guidelines and was approved by the Animal Ethics Committee of Islamic Azad University of Babol Branch, Babol, Iran.

**Fig. 2:**
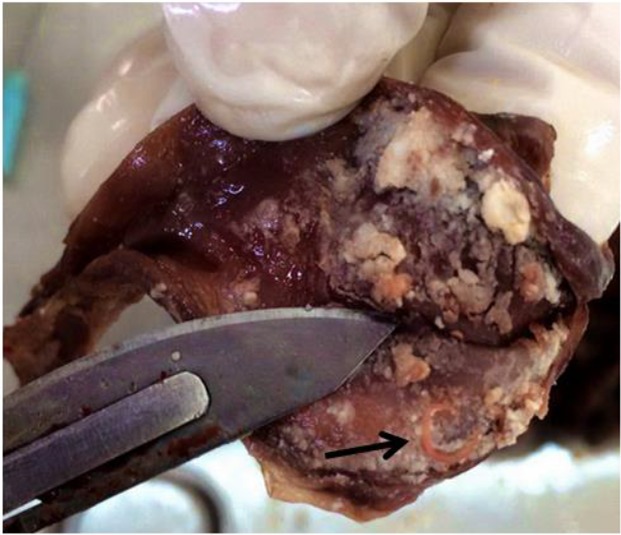
*S. lupi* in the esophageal mucosal membrane of a Sandy Fox (arrow)

**Fig. 3:**
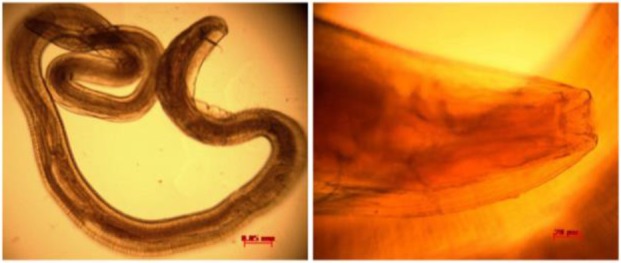
*S. lupi* Esophageal worm of Sandy Fox. Worms measure 55–75 mm

We found 9 nematodes of *S. lupi* approximately 55–75 mm long and spirally coiled with red-color blood in the esophageal (Figs. 2, 3).

On microscope examination, this nematode had the following features: trilobed lips and a short pharynx; the tail of a male species bears lateral alae, four pairs and one unpaired median pre-cloacal papillae and two pairs of post-cloacal papillae, with a group of minute papillae near the tail tip (9).

**Fig. 4:**
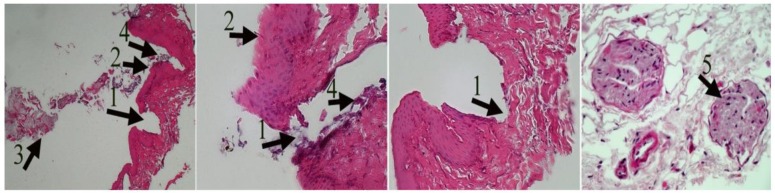
Esophageal tissue infected by *Spirocerca.* Epithelial destruction (1), Wall thickening (2), parasitic debris (3), Inflammatory cell infiltration (4), vascular thrombosis(5) H&E. ×40

Finally, the worms were identified as *S. lupi* based on the morphological characteristics of the adult parasites, their measurements, and their habitats, according to guidelines stipulated by Veterinary Parasitology (10). Histopathological study of the esophageal revealed interruption of the mucosa, and esophageal wall thickness. Microscopic examination has shown necrotic debris and remains of parasites in lumen, infiltration of inflammatory cells, necrosis, and vascular thrombosis (Fig. 4).

The formation of oesophageal nodules has taken 3 to 9 months to develop; however. It does not take long time to infect the animal.

## Discussion

This study presents *event*-related *potential S. lupi* infection and the larval immigration in the esophagus wall during the life cycle of *S. lupi* has been *well*-*described.*

Furthermore, similar cases of *S. lupi* were reported in stray dogs in Iran, involved 19.4% in Shiraz (11), 17.4% in Yasooj (12) as well as 17.9% of foxes in red in Sarab district, East Azerbaijan Province of Iran (13).

Matching to previous studies of canine spirocercosis, universal, outbreak varied from 10% to 85% based on necropsy outcome of vagabond dogs, outbreak of 23.5% in India (14), 40% in Bangladesh (15), 13% in South Africa (16), and 19% in Iran (11). Besides, In Kenya, an outbreak of 85% in vagabond dogs and 38% in owned dogs were recorded (17).

Nearly 25% of dogs infected with *S. lupi*, the esophageal nodules improve from inflammatory esophageal nodules to preneoplastic nodules and finally to sarcoma (18).

In Greece, 10% outbreak of *S. lupi* in owned dogs (19). The study in Grenada found 8.8% spirocercosis in owned dogs and 14.2% in vagabond dogs (20). Variety in outbreaks and severities of infection can undulate according to the climatic conversions.

Esophageal neoplasia, separated from sarcomas related to spirocercosis, was unusual in the Canine (21). Clearly, the effect of *S. lupi* on the health of canine necessarily increased knowledge of this parasite in the proximity of the infected region. In mutuality to clinical and pathological protests in canine, lesions of fox spirocercosis had just been published as gastric nodules, whereas slight lesions in the pectoral aorta were recorded in just one red fox and one gray fox in North America (22). The esophagus lesions were mostly granulomas. Therefore, it was not conventional, but sarcomas often can happen. These were fibrosarcoma and osteosarcoma, and their incidence rate increased considerably in native regions (23).

The cross-portion of the nodules was evaluated and generally showed a thick fibrotic wall, somewhat covered by epithelium, including a hole comprising worms and yellow fester (23,24). Spirocercosis was depended on a temperate up to intense native and systemic inflammatory responses. The systematic reaction in spirocercosis has proinflammatory appearance such as incremented circulatory, C-reactive protein, and IL-8. Proinflammatory reaction was forcefully depended on the pathophysiology of new bone formation (25). A study was done in Greece and *S. lupi* has been recorded in 0.4%–24.2% of the dogs except wild carnivores (6).

In this study, the efficacy of *S. lupi* in the esophagus of sandy fox has revealed wall thickening, interruption of the mucosa, and inflammatory cells infiltration. Spirocercosis was a serious status in native regions. In this research, we have studied the pathological transformation depended on *S. lupi* nematode in esophagus of sandy fox. As we know, this is the first report of this type of parasite and histopathological study in the sandy fox esophagus in Yazd Province, Iran.

## References

[1] Brito JC Acosta AL Álvares F Cuzin F. Biogeography and conservation of taxa from remote regions: An application of ecological-niche based models and GIS to North-African Canids. Biol Conserv. 2009; 142: 3020–3029.

[2] Harsini JI Rezaei HR Naderi S Moradi Hv. Phylogenetic status and genetic diversity of corsac fox (*Vulpes corsac*) in Golestan Province, Iran. Turk J Zool. 2017; 41: 250–258.

[3] Ferrantelli V Riili S Vicari D *Spirocerca lupi* isolated from gastric lesions in foxes (*Vulpes vulpes*) in Sicily (Italy). Pol J Vet Sci. 2010;13(3):465–71. 21033560

[4] Morandi F Angelico G Verin R Gavaudan S. Fatal spirocercosis in a free-ranging red fox. Vet Rec. 2014; 174: 228. 10.1136/vr.g1802 24578435

[5] Rinas MA Nesnek R Kinsella JM DeMatteo KE. Fatal aortic aneurysm and rupture in a neo-tropical bush dog (*Speothos venaticus*) caused by *Spirocerca lupi*. Vet Parasitol. 2009;164(2–4):347–9. 19515493 10.1016/j.vetpar.2009.05.006

[6] Diakou A Karamanavi E Eberhard M Kaldrimidou E. First report of *Spirocerca lupi* infection in red fox *Vulpes vulpes* in Greece. Wildlife Biol. 2012; 18: 333–336.

[7] Sasani F Javanbakht J Javaheri A Hassan MA Bashiri S. The evaluation of retrospective pathological lesions on spirocercosis (*Spirocerca lupi*) in dogs. J Parasit Dis. 2014;38(2):170–3. 24808646 10.1007/s12639-012-0216-y PMC4000364

[8] van der Merwe LL Kirberger RM Clift S *Spirocerca lupi* infection in the dog: a review. Vet J. 2008;176(3):294–309. 17512766 10.1016/j.tvjl.2007.02.032

[9] Taylor M A. Coop R L. Wall R L. 2007 Veterinary parasitology, 3rd ed. Oxford; Ames, Iowa: Blackwell Pub. 2007.

[10] Yamaguti S. Systema helminthum. In: The Nematodes of Vertebrates. Vol. 2 Interscience Publishers Inc, 1961; New York 679 p.

[11] Oryan A Sadjjadi SM Mehrabani D Kargar M. Spirocercosis and its complications in stray dogs in Shiraz, southern Iran. Vet Med Czech. 2008; 53 (11): 617–624.

[12] Moshfe A Mowlavi G Mobedi I Fauna of Zoontic Parasites of Stray Dogs in Yasouj Suburbs in 2008. Armaghane Danesh. 2011; 16 (1):80–89.

[13] Khanmohammadi M Fallah E Reyhani Rad S. Epidemiological studies on fauna and prevalence of parasite helminthes on red fox (*Vulpes vulpes*) in Sarab district, East Azerbaijan province, Iran. Ann Biol Res. 2011;2(5):246–51.

[14] Ramachandran PV Shakir SA Ramakrishnan R. Spirocercosis in canines: a necropsy survey. J Vet Sci Anim Husb. 1984; 13: 132–135.

[15] Shubhagata D Abdul A Mohammad MH Sikder S Muraduzzaman MM. Spirocercosis in stray dogs of Chittagong Metropolitan area of Bangladesh: an epidemiological and pathological investigation. Vet World. 2011; 4: 485–491.

[16] Minnaar WN Krecek RC Fourie LJ. Helminthes of dogs from a peri-urban resource-limited community in Free State Province, South Africa. Vet Parasitol. 2002;107(4):343–9. 12163245 10.1016/s0304-4017(02)00155-3

[17] Brodey RS Thompson RG Sayer PD Eugster B. *Spirocerca lupi* infection in dogs in Kenya. Vet. Parasitol. 1977; 3: 49–59.

[18] Dvir E Clift SJ Williams MC. Proposed histological progression of the *Spirocerca lupi*-induced oesophageal lesion in dogs. Vet Parasitol. 2010;168(1–2):71–7. 19963322 10.1016/j.vetpar.2009.10.023

[19] Mylonakis ME Koutinas AF Liapi MV A comparison of the prevalence of *Spirocerca lupi* in three groups of dogs with different life and hunting styles. J Helminthol. 2001;75(4):359–61. 11818054

[20] Chikweto A Bhaiyat MI Tiwari KP Spirocercosis in owned and stray dogs in Grenada. Vet Parasitol. 2012;190(3–4):613–6. 22841904 10.1016/j.vetpar.2012.07.006

[21] Murray M. Incidence and pathology of *Spirocerca lupi* in Kenya. J Comp Pathol. 1968;78(4):401–5. 4176219 10.1016/0021-9975(68)90037-6

[22] Al-Sabi MN Hansen MS Chriél M Genetically distinct isolates of *Spirocerca* sp. from a naturally infected red fox (*Vulpes vulpes*) from Denmark. Vet Parasitol. 2014;205(1–2):389–96. 25060226 10.1016/j.vetpar.2014.07.002

[23] Da Fonseca EJ Do Amarante EE Abboud LC Hees SJ Franco RJ Bruno JD. Fatal esophageal fibrosarcoma associated to parasitism by spirurid nematode *Spirocerca lupi* in a dog: a case report. J Parasit Dis. 2012; 36: 273–276. 24082542 10.1007/s12639-012-0108-1 PMC3427666

[24] Jones TC Hunt RD King NW. Disturbances of growth: aplasia to neoplasia. Vet. Pathol. 1997; 6: 81–112.

[25] Kirberger RM Clift SJ van Wilpe E Dvir E. *Spirocerca lupi*-associated vertebral changes: A radiologic-pathologic study. Vet Parasitol. 2013; 195(1–2):87–94. 23298568 10.1016/j.vetpar.2012.12.039

